# Preventive efficacy of Frontline^®^ Combo and Certifect^®^ against *Dipylidium caninum* infestation of cats and dogs using a natural flea (*Ctenocephalides felis*) infestation model

**DOI:** 10.1051/parasite/2013006

**Published:** 2013-02-19

**Authors:** Frederic Beugnet, Peet Delport, Hermann Luus, Dione Crafford, Josephus Fourie

**Affiliations:** 1 Merial 29 Av T. Garnier 69007 Lyon France; 2 ClinVet International (Pty) Ltd 9321 Universitas South Africa

**Keywords:** cats, dogs, *Ctenocephalides felis*, *Dipylidium caninum*, fipronil-(S)-methoprene, prevention

## Abstract

Two studies were performed to evaluate the effectiveness of two monthly topical anti-flea products for the prevention of *Dipylidium caninum* infestations in cats and dogs. A single treatment with Frontline^®^ Combo spot-on for cats (fipronil-(S)-methoprene) and two successive monthly treatments of Certifect^®^ for dogs (fipronil-amitraz-(S)-methoprene) were assessed for the prevention of *D. caninum* infestations following weekly challenges of treated cats or dogs with metacestode naturally-infected fleas. The rate of infestations using the model in cats versus dogs explains the choice of a 1-month trial in cats and a 2-month trial in dogs. The experimental flea-infection model resulted in a range of 22–53% of the fleas being infected by *Dipylidium* cysticercoids. The arithmetic mean flea counts recorded for the untreated cats ranged from 51.2 to 68. The geometric mean flea counts recorded for the Frontline Combo treated cats differed significantly (*p* < 0.05) from those of the untreated control cats on all assessment days. The arithmetic mean flea counts recorded for the untreated dogs ranged from 166.6 to 238.6. The geometric mean flea counts recorded for the Certifect treated dogs differed significantly (*p* < 0.001) from those of the untreated group on all assessment days. Frontline Combo treatment on cats provided ≥99.8% persistent anti-flea efficacy throughout the 30-day treatment period. In the dog study, the two Certifect treatments provided ≥97% persistent efficacy throughout the 60-day study. Based on the collection of expelled *D. caninum* proglottids by cats, 100% (6/6) of the control cats and 0% (0/6) of Frontline Combo treated cats were infested with *D. caninum*. Frontline Combo spot-on for cats was therefore 100% effective in preventing infection with *D. caninum*. In dogs, 7 out of the 8 control group dogs (87.5%) produced proglottids following infestation of infected fleas, whereas 0 out of 8 dogs (0%) in the treated group were infected. The infection rates of the two groups were significantly different. The percent effectiveness for the Certifect treatment group for the prevention of *D. caninum* infection was 100% during this 2-month trial. No treatment-related adverse events were observed in either cats or dogs during these studies.

## Abstract (fr)

Deux études ont été effectuées pour évaluer l’efficacité de deux produits mensuels anti-puces pour la prévention de l’infestation de *Dipylidium caninum* chez les chats et chiens. Un traitement unique avec Frontline^®^ Combo spot-on pour chats (fipronil-(S)-methoprene) et deux traitements mensuels successifs de Certifect^®^ pour chiens (fipronil-amitraz-(S)-methoprene) ont été évalués vis-à-vis de la prévention de l’infestation par *D. caninum* suivant des infestations hebdomadaires par des puces naturellement infectées par les larves du cestode. Le taux des infestations utilisant le modèle chez les chats, par rapport aux chiens, explique le choix d’un essai sur un mois chez les chats et de deux mois chez les chiens. Le modèle expérimental d’infection des puces a abouti à un taux d’infection de 22 à 53 % des puces par les larves cysticercoïdes de *Dipylidium*. Le comptage sur les chats non traités a donné une moyenne géométrique de 51.2 à 68 puces par chat. Les moyennes géométriques du nombre de puces comptabilisées sur les chats traités par le Frontline Combo étaient significativement différentes (*p* < 0,05) des chats non traités pour tous les jours de comptage. La moyenne géométrique du nombre de puces sur les chiens non traités allait de 166,6 à 238,6. Les moyennes géométriques du nombre de puces sur les chiens traités au Certifect étaient significativement différentes des chiens non traités pour tous les jours de comptage (*p* < 0,001). Le traitement des chats avec le Frontline Combo a eu une efficacité anti-puces persistante ≥99,8 % durant la période d’étude de 30 jours. Dans l’étude des chiens, les deux traitements avec le Certifect ont abouti à une efficacité persistante ≥97 % durant les 60 jours. Sur la base de la collecte des segments de *D. caninum* rejetés par les chats, 100 % (6/6) des chats non traités et 0 % (0/6) des chats traités avec le Frontline Combo ont été infestés par *D. caninum*. Frontline Combo spot-on a donc été efficace à 100 % dans la prévention de l’infestation des chats par *D. caninum*. Chez les chiens, 7 des 8 chiens non traités (87,5 %) ont produit des proglottis suivant l’infestation par les puces infectées, tandis qu’aucun des 8 chiens traités n’a été infesté (0 %). Les taux d’infestation étaient significativement différents entre les deux groupes. L’efficacité du traitement avec le Certifect dans la prévention de l’infestation des chiens par *D. caninum* a été de 100 % durant les 2 mois de l’étude. Aucun effet indésirable lié aux traitements n’a été noté durant ces études, ni chez les chats ni chez les chiens.

## Introduction

The cat flea, *Ctenocephalides felis*, is widespread all over the world [1, 4, 9, 10, 12, 13, 15]. Its low specificity explains its common infestations of cats, and dogs, and many other mammals including human beings. Flea infestations pose serious health problems to cats and dogs such as itching, hair loss, flea allergy dermatitis, and increase the risk of transmitting numerous pathogens including *Bartonella henselae* and *Rickettsia felis* [3, 14, 22]. The cat flea is also the intermediate host of a tapeworm, *Dipylidium caninum* [6]. Larval fleas ingest eggs of *D. caninum*; the eggs hatch; and the hexacanth embryos infect the flea larva and develop with the flea. Once the adult flea emerges and infests a host, the hexacanth develops into an infective cysticercoid (metacestode stage) in the flea within 2–3 days. Carnivores become infested through the ingestion of infected fleas containing cysticercoid larvae, and adult *D. caninum* typically begin shedding proglottids within 2–3 weeks. *Dipylidium caninum* is common worldwide, infesting both cats and dogs, and is zoonotic, even if human infestations are really scarce [5, 8, 19].

As such, the regular use of an effective pulicidal compound is important to protect cats and dogs against the various detrimental effects of flea infestations. Many insecticides and/or acaricides are available for use in cats and/or dogs in a variety of formulations [2]. It can be assumed that the risk of pathogen transmission from arthropods to their hosts may be significantly decreased if fleas or ticks are killed quickly enough (in <24 h) and if this speed of kill is sustained during a long period of time (>1 month [2]). Several authors have studied the indirect preventive role of insecticidal and/or acaricidal treatments against the transmission of flea or tick-borne diseases [7, 16–18, 20]. However, essentially all of the flea or tick transmitted pathogens studied were bacteria or protozoa: *Ehrlichia canis*, *Anaplasma phagocytophilum*, *Borrelia burgdorferi*, *B. henselae*. Except for the paper concerning *Bartonella* [7], the majority of these studies were conducted in dogs to assess the prevention of tick-borne pathogens. A recent paper has demonstrated that indirect protection also could be afforded by preventing *D. caninum* transmission in cats [11]. In this paper, the prophylactic protection was conferred by the anti-flea and -tick collar Seresto™ (imidacloprid 10% w/w – flumethrin 4.5% w/w in a slow-release matrix collar, Bayer).

Fipronil is one of the leading insecticide and acaricide products used to protect pets against both flea and tick infestation. It is used alone or in combination with other active ingredients in topical formulations [2]. Based on its speed of kill (24 h for fleas, 48 h for ticks if used alone and 24 h if used in combination with amitraz) and long-lasting effect during at least 1 month [26], it was hypothesised that its regular use may provide a protective effect against *D. caninum* transmission for both cats and dogs.

The objective of the two studies reported here was to evaluate the effectiveness of monthly topical anti-flea products for the prevention of *D. caninum* infestations in cats and dogs. A single treatment of Frontline Combo spot-on for cats and two successive monthly treatments of Certifect for dogs were assessed for the prevention of *D. caninum* infections following weekly challenges with *D. caninum* infected, newly emerged fleas applied to treated cats or dogs [21, 26]. Due to the infestation model itself using infected fleas, the rate of animal infestation with fleas varies between dogs and cats, justifying a 1-month and a 2-month study for cats and dogs, respectively. This is due to the natural grooming behaviour of cats and their capacity to ingest many fleas in a short time, which is not at all the case for dogs. This “ingestion rate” also explains why more fleas were used to infest dogs compared to cats.

## Materials and methods

These two studies followed a single centre, controlled efficacy, randomised parallel group design.

### Production of fleas infected by *Dipylidium* metacestodes

To assess the prophylactic effect of anti-flea treatment, it was first necessary to produce *Dipylidium*-infected fleas. In order to do so, donor cats infested with *D. caninum* were infested with fleas. These cats were placed in individual cages. Flea eggs and shed proglottids were collected in a paper-covered pan below the cages every 24 or 48 h. The contents were sieved to remove gross debris, such as hair. The sieved material containing flea eggs, *Dipylidium* proglottids and egg packets, were placed in an incubator (at 24 to 28.5 °C) in Petri dishes. The flea eggs started hatching after approximately 3 days, and larvae were maintained only with the sieved material for another 2 days (i.e., up to ~5 days after sieving). Then the mixture of larvae, proglottids and egg packet was transferred into classic flea breeding medium, made of a mixture of sand and crushed dried cat food, to ensure that the larvae could feed and develop adequately.

The development of *Dipylidium* larval stages can be divided into two steps. The first one is the metacestode development in the flea larvae, pupae and newly emerged fleas [23, 24]. This maturation of the metacestodes can be discerned by their morphology changes, as originally described by Venard [25]. The second step is the final maturation in adult fleas that have infested their host. A preliminary assessment done by Pugh and Moorhouse showed that fleas typically are found to be infective for cats at 14–16 days after the pupae were sieved from the medium [23, 24].

### Design of the studies

The cat study was conducted on two groups of six cats each: Group 1 was an untreated control and Group 2 consisted of cats treated with Frontline Combo spot-on (named Frontline Plus for cats in some countries).

The study followed a randomised block design. The 12 cats included were ranked within gender in descending order of individual pre-treatment flea counts using uninfected fleas. Animal IDs were used as the criteria to break any ties in pre-treatment flea counts. Animals were blocked into blocks of two cats each. Within each block, cats were randomly allocated to Groups 1 or 2. The study was not blinded. The cats were domestic cats aged 6 months or more, weighing 1 kg or more (Table 1). They were healthy at the date of start and had not been treated with a topical or systemic acaricide/insecticide for 12 weeks prior to Day 0.

**Table 1.** Description of the animals included in the studies.

Control cats			Treated cats				
ID	G	BW (Day-2)	ID	G	BW (Day-2)		
DF6 952	F	2.48	CC3 B65	F	3.61		
CD4 302	F	2.9	DF7 E10	F	2.28		
CD5 1E7	F	3.28	6BE 343	F	3.16		
EA0 4FE	F	3.44	CD1 47C	F	2.74		
E49 9C5	M	3.33	CC3 735	M	3.09		
CC0 55B	M	3.31	CD5 716	M	4.17		
Mean		3.18	Mean		3.25		
Control dogs				Treated dogs			
ID	G	BW (Day-1)	BW (Day+29)	ID	G	BW (Day-1)	BW (Day+29)
CC4 90E	F	17.74	17.81	CBD D00	F	15.60	14.93
E16 E41	F	14.68	15.32	8B3 7C1	F	14.70	14.63
E49 BE7	F	13.62	14.02	DF6 707	F	13.05	13.36
E44 A95	F	12.90	13.12	DF7 CEB	F	12.76	12.63
DFA 390	F	11.34	11.10	DF4 CC6	F	12.08	12.13
CC0 FE7	F	10.88	10.52	8B1 6BB	F	11.28	10.71
CD3 EC5	F	10.52	9.94	CDB DAE	F	9.08	8.42
956 7EC	M	14.48	15.89	DF5 A68	M	18.24	19.09
Mean		13.27	13.47	Mean		13.35	13.24

ID = identification number; G = gender; BW = body weight (kg).

The Group 2 cats were treated each with one 0.5 mL pipette of the combination fipronil – (S)-methoprene (Frontline Combo spot-on cats) at Day 0 following label recommendations.

The coat on the back of the cat at the base of the neck and in front of the shoulder blades was parted until the skin was visible. The treatment then was administered by placing the tip of the opened pipette on the skin and squeezing the pipette several times to empty its contents completely and directly onto the skin in one spot.

Each cat was infested with 100 newly emerged adult fleas; that had been exposed to *D. caninum* as larvae on Days 0, 7, 14, 21 and 28 (Table 2). At 48 h (±2 h) following each infestation, flea efficacy was assessed. Each cat was flea-combed; counts were performed and recorded; then, any collected fleas were reapplied to the respective cat once the flea count was completed except at Day 30 where fleas were removed definitely. Assessments for *Dipylidium* infection as well as daily observations for proglottid production began at Day 21 and continued for animals remaining negative through Day 60.

**Table 2.** Summary of the schedule of operations.

Operation	Cats	Dogs
Acclimatisation	Days −14 to −1	Days −7 to −1
Flea infestation with 100 non infected fleas for randomisation purposes	Day −11	Day −7
Ranking and allocations to groups	Days −3 or −2	Days −3 or −2
Flea infestations with 100 (cats) or 250 (dogs) fleas from a population of *D. caninum* infected fleas	Days 0, +7, +14, +21 and +28	Days 0, +7, +14, +21,+28, +35, +42, +49, +56
Administration of treatment	Day 0	Day 0
Flea counts^1^	Days −9, +2, +9, +16, +23 and +30	Days −6, +2, +9, +16, +23, +30, +37, +44, +51, +58
Monitoring for expelled *D. caninum* proglottids	Days −14; −1; and daily from Day +21 to Day +60	Days −1, and then Daily from Day +21 to Day +86

1 Fleas (except on Days −5 and −10) were counted and placed back on the cats and dogs 48 ± 2 h post-infestation.

The dog study was conducted using two groups of eight dogs each: Group 1 was an untreated control and Group 2 consisted of dogs treated with the combination fipronil, amitraz, (S)-methoprene (Certifect spot-on dogs). The study followed a randomised block design. The 16 dogs included were ranked in descending order of individual pre-treatment flea counts with uninfected fleas. Animals were blocked into blocks of two dogs each. Within blocks, dogs were allocated randomly to the groups. The dogs were mixed breeds, males and females, aged 6 months and older, weighing 9.08 to 20.84 kg (Table 1). They were healthy at the date of allocation (Day-14) and had not been treated with any topical or systemic acaricidal/insecticidal products for at least 12 weeks before the treatment date (Day 0). At Day 0 and Day 30, the Group 2 dogs were treated with commercially available Certifect for dogs following the label and dose recommendations. The coat on the back of the dog at the middle of the neck and secondly at the base of the neck was parted until the skin was visible. The treatment was administered by placing the tip of the opened pipette on the skin and squeezing the pipette to deliver the full dose onto the skin in two spots (as clearly indicated on the packaging and label insert).

Each dog was infested with 250 newly emerged adult fleas that had been exposed to *D. caninum* as larvae on Days 0, 7, 14, 21, 28, 35, 42, 49 and 56 (Table 2). At 48 h (±2 h) following each infestation, flea efficacy was assessed. Each dog was flea-combed; counts were performed and recorded; then, any collected fleas were reapplied to the respective dog once the flea-count was completed except at Day 60 where the fleas were definitely removed. Assessments for *Dipylidium*-infection as well as daily observations for proglottid production began at Day 21 and continued for animals remaining negative through Day 86. The dog study was conducted over a period of 2 months, because preliminary investigations showed that the success of dog *Dipylidium* infestation by potentially infected fleas was lower than in cats. For the same reason, the flea challenges were higher in dogs than in cats.

In both studies, the animals (cats and dogs) were kept individually in runs during the entire study period. No contact between animals was possible. The animals were exposed to ambient temperature and lighting was provided by natural sunlight. Each animal was identified individually and assigned to a specific, individually-identified housing unit throughout the study.

All the animals were observed daily from Day −14 to Day 60 (cats) or Day 86 (dogs) for general health, and treated cats and dogs were observed hourly for 4 h immediately post-treatment for possible adverse events.

For all post Day 0 treatment flea infestations, the same laboratory bred strain (ClinVet European strain) of *C. felis* infected with a South African *D. caninum* strain was used. Prior to each post-treatment infestation, the *D. caninum* infection rate for the fleas was determined by microscopically examining 100 fleas for *D. caninum* cysticercoids. The prevalence of *D. caninum* infection in the weekly flea batches used, ranged from 31 to 43% in the cat study and 22 to 53% in the dog study.

The experimental unit was designed in compliance with the South African National Standard “SANS 10386:2008 *The care and use of animals for scientific purposes*”. The protocols were submitted to the Clinvet Animal Ethics Committee (CAEC) as well as Bloemfontein University. After approval, a certificate was issued authorising the test facility to conduct the studies. Members of the CAEC had the authority to inspect the test facility and the animals at will. The studies were performed under GCP (Good Clinical Practices) rules.

### Monitoring for expelled *D. caninum* proglottids

Cat and dog faeces were screened during acclimatisation and daily from Day 21 to Day 60 (cats) or 86 (dogs) to detect expelled proglottids. This screening involved a primary visual, macroscopic observation to detect proglottids in freshly shed faeces or around the anal and perineal region of animals, in their cages or on hairs. In the second step, after macroscopical observation, freshly shed faeces were washed through sieves (aperture size 0.3 mm). The residues of the sieves were suspended in a small amount of water that were examined macroscopically for the presence of tapeworm proglottids. No flotation technique was used given its poor sensitivity to detect cestode proglottids compared to sieving technique using full fecal material [6]. *Dipylidium* eggs were not searched by coproscopy as they are rarely present in dog and cat faeces [6]. All proglottids or worm fragments that were found were finally examined microscopically for proper identification with proglottids, preserved individually in identified vials of formalin and maintained through the end of the study as a physical record of the diagnosis. Once an individual cat or dog was diagnosed positive for proglottids or eggs of *D. caninum* on two separate occasions, no further faecal examinations were conducted for that animal.

### Methods for calculating the product efficacy for preventing tapeworm infection (primary criteria)

The primary assessment variable was the presence or absence of *D. caninum* infections in cats and dogs. The percentage efficacy for the prevention of *D. caninum* infection in the treatment group was calculated at the end of each study as follows:

Efficacy (%) = 100 × (*T*_c_ − *T*_t_)/*T*_c_, where:
*T*_c_ = Total number of infected cats in the negative control Group 1;
*T*_t_ = Total number of infected cats in the treatment Group 2.

### Methods for calculating the adulticidal product efficacy (secondary criteria)

The 48-h efficacy against fleas for the treatment group was calculated on each assessment day. Both geometric and arithmetic means were calculated. The insecticidal efficacy was calculated based on the geometric means.

Efficacy against fleas was calculated according to the following formula:

Efficacy (%) = 100 × (*m*_c_ − *m*_t_)/*m*_c_, where:
*m*_c_ = geometric mean of live fleas on the negative control group (Group 1)
*m*_t_ = geometric mean of live fleas on the treated group (Group 2)

### Comparison between groups

The study groups were compared with regard to the flea counts and *D. caninum* infection rates. With respect to the flea counts, a one-way ANOVA test was used. SAS^®^ version 8 was used for all the statistical analyses. The level of significance of the formal tests was set at 5%; all tests were two sided.

## Results

### Flea counts

Arithmetic and geometric mean flea counts on the various assessment days for both study groups are summarised in Tables 3 (cats) and 4 (dogs). The arithmetic mean flea counts recorded for the untreated cats ranged from 51.2 to 68. The geometric mean flea counts recorded for the Frontline Combo treated cats differed significantly (*p* < 0.05) from those of the untreated cats on all assessments.

**Table 3.** Mean flea counts and insecticidal efficacies in cats.

Day	Group 1 – Untreated cats		Group 2 – Frontline^®^ Combo treated cats	
	Arithmetic mean [min–max/*SD*]	Geometric mean^1^	Arithmetic mean [min–max]^2^	Geometric mean^1^,^2^
2	58.5 [35–81/58.5]	56.5	0 [0–0/0] (100%)	0 (100%)
9	64.8 [24–110/64.8]	58.6	0 [0–0/0] (100%)	0 (100%)
16	68.0 [25–121/68]	61.4	0 [0–0/0] (100%)	0 (100%)
23	51.2 [28–95/51.2]	47.4	0 [0–0/0] (100%)	0 (100%)
30	61.3 [42–105/61.3]	58.7	0.2 [0–1/0.4] (99.7%)	0.1 (99.8%)

1 Group 2 differed statistically significantly (*p* < 0.05) from the untreated control Group 1 on all post-treatment assessment days.

2 % of efficacy (XX%).

*SD* = standard deviation.

**Table 4.** Mean flea counts and insecticidal efficacies in dogs.

Day	Group 1 – untreated control		Group 2 – Certifect^®^ treated dogs	
	Arithmetic mean [min–max/*SD*]	Geometric mean^1^	Arithmetic mean [min – max/*SD*]^2^	Geometric mean^1^,^2^
2	189.0 [150–227/25]	187.5	0 [0–0–/0] (100%)	0 (100%)
9	218.8 [107–302/61]	210.1	0 [0–0–/0] (100%)	0 (100%)
16	238.6 [106–405 /91.6]	222.9	0 [0–0–/0] (100%)	0 (100%)
23	237.3 [101–424/120]	210.3	0 [0–0–/0] (100%)	0 (100%)
30*	212.0 [101–387/87.8]	197.1	8.6 [0–20/7] (95.9%)	5.8 (97%)
37	208.6 [123–324/78.1]	196.2	0 [0–0–/0] (100%)	0 (100%)
44	189.6 [79–323/94]	169.7	0 [0–0–/0] (100%)	0 (100%)
51	166.6 [85–320/76.1]	152.7	1.1 [0–9/3.2] (99.3%)	0.3 (99.8%)
58	175.0 [101–325/71.1]	164.3	12.1 [0–87/30.3] (93.1%)	2.0 (98.8%)

1 Group 2 differed statistically significantly (*p* < 0.001) from the untreated control Group 1 on all post-treatment assessment days.

2 % of efficacy (XX%).

* Retreatment at Day 30 after flea count.

The arithmetic mean flea counts recorded for the untreated dogs ranged from 166.6 to 238.6, indicating heavy weekly flea challenges. The geometric mean flea counts recorded for the Certifect treated dogs differed significantly (*p* < 0.001) from those of the untreated dogs on all assessment days.

Frontline Combo treatment on cats provided ≥99.8% persistent efficacy for the 30 days. In dogs, the two Certifect treatments provided ≥97% persistent efficacy during the 60 days.

### *Dipylidium caninum* counts

Expelled *D. caninum* proglottids were observed in 100% (6/6) of the control cats and 0% (0/6) of Frontline Combo treated cats. Frontline Combo spot-on was 100% effective in preventing infection with *D. caninum* following a single treatment and weekly flea infestations without any flea removal (Table 5). In dogs, 7 out of 8 dogs (87.5%) in the control group and 0 out of 8 dogs (0%) in the treated group were infected with *D. caninum* (Table 6). The difference between the two groups was significant (*p* = 0.0004). The percent efficacy for the Certifect treatment group for the prevention of *D. caninum* infection was 100% during this 2-month trial facing heavy weekly infestations.

**Table 5.** *Dipylidium caninum* proglottid collections in cats following daily search.

Group 1 – Negative control		Group 2 – Frontline^®^ Combo treated cats	
Cat ID	*D. caninum* proglottids observed*	Cat ID	*D. caninum* proglottids observed
DF6 952	Days +22 and +28	CC3 B65	None observed in 39 exams
CD4 302	Days +28 and +29	DF7 E10	None observed in 39 exams
CD5 1E7	Days +28 and +29	6BE 343	None observed in 39 exams
EA0 4FE	Days +26 and +27	CD1 47C	None observed in 39 exams
E49 9C5	Days +27 and +28	CC3 735	None observed in 39 exams
CC0 55B	Days +26 and +28	CD5 716	None observed in 39 exams
Percent infested	100%	Percent infested	0%

* Search for *Dipylidium* proglottids was conducted daily from Day 21 to Day 60. Once an individual cat was diagnosed positive for proglottids of *D. caninum* on two separate occasions, no further faecal examinations were conducted for that animal who was considered as positive.

**Table 6.** *D. caninum* proglottid collections in dogs following daily search

Group 1 – Untreated dogs		Group 2 – Certifect^®^ treated dogs	
Dog ID	*D. caninum* proglottids observed*	Dog ID	*D. caninum* proglottids observed
CC4 90E	Days +59 and +60	CBD D00	None observed in 65 exams
E16 E41	Days +28 and +29	8B3 7C1	None observed in 65 exams
E49 BE7	Days +59 and +61	DF6 707	None observed in 65 exams
E44 A95	Days +30 and +31	DF7 CEB	None observed in 65 exams
DFA 390	Days +28 and +29	DF4 CC6	None observed in 65 exams
CC0 FE7	Days +41 and +43	8B1 6BB	None observed in 65 exams
CD3 EC5	None observed	CDB DAE	None observed in 65 exams
956 7EC	Days +29 and +30	DF5 A68	None observed in 65 exams
Percent infested	87.50%	Percent infested	0%

* Search for *Dipylidium* proglottids was conducted daily from Day 21 to Day 86. Once an individual dog was diagnosed positive for proglottids of *D. caninum* on two separate occasions, no further faecal examinations were conducted for that animal who was considered as positive.

No adverse events were observed in either cats or dogs during each study.

## Discussion

The persistent flea control provided by the treatments in each of the two studies was in accordance with their respective available published data and labelling [21, 26].

The experimental flea-infection model worked well, producing a population of *Dipylidium* cysticercoid-infected fleas at a rate of 22–53%. This model allows an effective option for studies intending to assess treatment and prevention of fleas and tapeworms in cats or dogs. The natural infection rate of fleas seems to be very low (max of 1%) based on the literature data. It highlights the protective efficacy obtained during these challenges, which are far higher than the natural risk [5, 6, 23, 25]. Frontline Combo and Certifect were 100% effective in preventing infestations with *D. caninum* in cats and dogs, respectively, despite the fact that fleas were reapplied on the animals until Day 30 for cats and Day 60 for dogs. Typically, animals infested with fleas will groom themselves and ingest fleas, and cats are particularly adept groomers. In order to provide protection from *D. caninum* infection, the anti-flea treatment needs to kill fleas before the maturation of the cysticercoid. Based on available data, it seems that cysticercoid larvae need at least 24–36 h to become infective for the definitive host [11, 23, 24]. This development is temperature related. A body temperature >30 °C seems to induce this maturation. We can assume that by killing fleas within 24 h, even if killed fleas are ingested, no infection can occur.

In these controlled studies, the protection was complete, but controlled exposure and perfect compliance do not always occur under field conditions. Compliance with any medication or treatment by pet owners can be highly variable, potentially allowing some infected fleas to survive, allowing the cysticercoid to mature and infect their hosts [10]. It is important to keep in mind that if infected fleas are present, it is likely that pet or feral animals with access to the home or yard (garden) are infested with *Dipylidium* too. In that sense, the best protective measure is to combine regular anti-flea treatments, good observation and appropriate deworming.

Certifect^®^ and Frontline are registered trademarks of Merial. All other marks are the property of their respective owners.

### Conflict of interest

The first author is an employee of Merial, which produced the veterinary drugs used in these studies.
